# Properties and Applications of Advanced Textile Materials

**DOI:** 10.3390/ma19122513

**Published:** 2026-06-11

**Authors:** Fujuan Liu

**Affiliations:** National Engineering Laboratory for Modern Silk, College of Textile and Clothing Engineering, Soochow University, 199 Ren-Ai Road, Suzhou 215123, China; liufujuan@suda.edu.cn

## 1. Introduction

Advanced textile materials have undergone a revolutionary transformation from traditional clothing and decorative fabrics to high-performance, intelligent, and environmentally friendly functional systems that connect materials science, fiber engineering, wearable electronics, energy efficiency, and sustainable technologies. In addition to basic coverage and protection, modern advanced textiles also integrate complex structural design, stimulus response characteristics, multifunctional synergy, and scalable manufacturing, making them indispensable in personal healthcare, outdoor protection, building energy efficiency, ocean utilization, aerospace, and flexible electronic products. This Special Issue, titled “Performance and Applications of Advanced Textile Materials”, brings together frontier research with a focus on structural innovation, performance tuning, multifunctional integration, and practical applications. Based on these contributions, this editorial systematically reviews the frontier progress, core design strategies, typical functional characteristics, and application prospects of advanced textile materials, and provides prospects for future development.

Biomimetic and multi-scale structural design have become the most powerful strategies to overcome the performance bottleneck of traditional textiles [1,2,3]. By mimicking the micro/nano structures and functional mechanisms of organisms such as chameleons, plant leaves, animal skin, and polar bear hair, researchers have endowed textiles with distinguished optical, thermal, mass transfer, and mechanical properties. Inspired by the chameleon’s thermochromic thermoregulation, Zhang et al. [1] developed a thermochromic phase-changing aerogel fiber via a one-step electrospinning process, which realized the autonomous switching between solar heating and radiation cooling modes without any external intervention. This material exhibits high latent heat (154.7 J·g^−1^), ultra-low thermal conductivity (24.8 mW·m^−1^·K^−1^), and combines ultra-light, elastic, flame-retardant, and washable properties. Cheng et al. [2] proposed a breathable dual-mode leather-like nanotextile with an asymmetric photon wrinkle structure, which can achieve switchable high-efficiency radiation cooling and solar heating by flipping, greatly expanding the human thermal comfort zone to 44.1 °C. Hu et al. [3] simulated leaf stomata and a three-layer leaf vein structure to construct a two-dimensional photothermal fabric for solar-driven seawater desalination, achieving four-plane evaporation and effective salt resistance, with evaporation rates of up to 2.6 kg·m^−2^·h^−1^ and 4.2 kg·m^−2^·h^−1^ under wind assistance. These biomimetic strategies simultaneously enhance heat transfer, light modulation, and steam diffusion, providing a general paradigm for high-performance textile design [4,5].

Thermal management textiles represent the most active and application-oriented direction in advanced textiles, achieving a breakthrough from passive insulation to zero energy adaptive regulation [1,2,3,6,7,8,9,10,11,12,13]. The core lies in precise spectral modulation, phase change energy storage, and thermal insulation engineering. Liang et al. [7] developed a sandwich-structured radiation cooling fabric embedded with bead-shaped nanofibers and large-sized SiO_2_ particles, achieving ultra-high solar reflectivity (97.8%) and infrared emissivity (98.7%), providing an average cooling effect of 9 °C and excellent UV protection (UPF = 132). In response to the esthetic needs of wearable scenarios, Zhu et al. [6] developed an invariant color thermal fabric based on narrow-bandgap organic semiconductors. The fabric absorbs near-infrared light for heating while maintaining its original color, with a temperature rise of 4–8 °C within 5 min and an antibacterial rate exceeding 95.3%. Overall, these studies have established a complete design system of “biomimetic inspiration spectral regulation multi-scale structure functional integration” for personal thermal management in all seasons, providing low-carbon solutions for building energy conservation and outdoor comfort [8,9,11,14].

Flexible electronic textiles are accelerating the commercialization of next-generation wearable systems, enabling textiles to simultaneously perform mechanical support, sensing, energy harvesting, and physiological monitoring [15,16,17,18,19,20]. By combining polymers with carbon nanotubes MXene, Graphene, conductive polymers, and organic semiconductors, stable photothermal, piezoelectric, thermoelectric, pressure-sensitive, and electrothermal properties in textiles are achieved, while maintaining high breathability, moisture permeability, and washability [3,4,6,17]. The energy harvesting technology based on textiles, including frictional electric nanogenerators, thermoelectric fabrics, and solar-driven steam generators, has been widely studied, achieving self-powered wearable devices without the need for external batteries [3,7,21,22,23,24]. These flexible electronic textiles have shown great potential in health monitoring, human–computer interaction, sports rehabilitation, and marine resource utilization [16,18,25]. Unlike traditional rigid electronic products, textile-based systems perfectly fit the human body, providing long-term comfort and reliability.

Multifunctional collaboration has become an important feature of advanced textiles for adapting to complex application scenarios [1,4,5,6,7,14,26]. Given that single functional textiles can no longer meet evolving practical needs, the field is undergoing rapid development in multiple promising directions, including blast protection, smart textiles, biomimetic manufacturing, and environmental remediation. This editorial mainly summarizes the latest research progress in material design, performance optimization, and mechanism exploration. Additionally, this Special Issue focuses on properties and applications of advanced textile materials, collecting eight research articles and two reviews. The relevant research highlights of these works are presented in the sections below.

## 2. An Overview of Published Articles

Yin et al. (contribution 1) carried out a systematic investigation into the formation mechanism and motion characteristics of multiple jets during spherical section free surface electrospinning (SSFSE), aiming to achieve efficient and scalable preparation of nanofibers. They established a mechanical model for multi-jet behavior and confirmed that electric field intensity acts as the core factor dominating jet initiation and motion, while establishing the quantitative relationship between jet initial velocity and electric field distribution on the solution surface. By combining the magnetohydrodynamic (MHD) model, RNG k–ε turbulence model and volume of fluid (VOF) model, they numerically simulated the jet motion under spinnerets with different spherical radii. The results indicated that a larger sphere radius reduces the maximum jet velocity but expands the effective jet generation area and intensifies inter-jet interaction, leading to more obvious outward expansion of the jet trajectory. The optimal spherical radius was determined to be 75 mm, which achieves a good balance between fiber yield and diameter uniformity. Experiments using a low-conductivity TPU solution verified the simulation results with an accuracy of about 92.3%. This work provides a theoretical basis for increasing jet density per unit area and realizing continuous, uniform and high-efficiency production of micro/nanofibers.

Pamela Miśkiewicz’s article (contribution 2) designed and evaluated two four-layer textile composites coated with ZrO_2_/Al bilayers via PVD magnetron sputtering, intended for use in heat-protective gloves for metallurgical workers. The composites are composed of basalt fabric, silicone sealant, Mylar^®^ foil and ZrO_2_/Al coatings of different thicknesses. High-resolution X-ray micro-CT was used to characterize the layer structure, porosity and coating uniformity, showing that the two composites have a highly consistent structure and porosity with a difference of less than 1%. Thermal performance tests were conducted according to European standards under contact heat, radiant heat and flame heat. Both composites reached Level 1 for contact heat protection at 100 °C, Level 4 for radiant heat protection and Level 3 for flame heat protection. Composite β with a thicker coating exhibited better thermal insulation. The results confirm that these composites are suitable for the back part of high-temperature protective gloves and provide reliable heat insulation under severe working conditions.

The article by Matusiak et al. [27] is an in-depth study on liquid sweat transport in combined assemblies of firefighter underwear and multilayer protective clothing, to improve the thermophysiological comfort of firefighters. They tested four kinds of knitted underwear fabrics together with four commercial multilayer firefighter clothing systems using the MMT290 Moisture Management Tester. The results show that pure underwear fabrics have good liquid sweat transport capacity, while the complete multilayer protective clothing blocks liquid penetration and traps sweat inside the inner layer. The overall moisture management capacity (OMMC) and one-way transport index (R) of the assembled system are highly dependent on material matching. The combination of the inner layer of the S3 multilayer system and F2 pique underwear presents the best liquid transport performance. The specially designed firefighter underwear F4, despite poor individual performance, achieves good sweat management when matched with suitable inner layers while providing flame-retardant and antistatic functions. This study proves that evaluating assembled systems rather than individual materials is essential for optimizing sweat management in firefighter clothing.

Liu et al. [28] developed tyrosinase-catalyzed crosslinked silk fibroin (c-SF) membranes and precisely regulated their degradation properties for application in superficial wound repair. The crosslinking degree was tailored by adjusting the TYR/SF ratio, reaching up to 88.17 ± 0.20% at a ratio of 20/6000 without altering the crystal structure. In vitro degradation tests revealed that all membranes remained stable in pure PBS solution, while exhibiting controllable degradation in the presence of collagenase. As the TYR/SF ratio increased, the 7-day residual mass rose from 23.31 ± 1.35% to 60.12 ± 0.82%, accompanied by a slight increase in the β-sheet structure and a reduction in the release of free amino acids. CCK-8 and protein synthesis assays confirmed excellent cytocompatibility with L929 cells. The degradation products containing stable free amino acids were safe to surrounding tissues and could even promote wound healing. This work provides a green, enzymatically crosslinked SF membrane with tunable rapid degradability, offering a promising candidate for biodegradable wound repair materials.

The article by Weiter et al. (contribution 3) focuses on chemical modification of conventional glass fiber filter media using targeted surface-active agents, aiming to synergistically improve separation efficiency, reduce differential pressure (ΔP), and enhance dirt holding capacity (DHC). Surface energy was quantified via contact angle measurements, and a work of adhesion model was established to predict interactions between contaminants, filter media, and base fluid. Three surface modifications were prepared: hydrophilic plasma treatment (S-C1), hydrophilic PVA/PVB coating (S-C2), and hydrophobic fluorocarbon coating (S-C3). Textile characterization confirmed that basic physical properties, including thickness, air permeability, and pore size distribution, remained unchanged. ISO 16889 [29] multi-pass filtration tests showed that hydrophilic modifications significantly improved removal efficiency for fine particles below 7 μm, while the hydrophobic treatment reduced efficiency. Differential pressure was unaffected by hydrophilic treatments but notably increased under hydrophobic modification. DHC slightly decreased for all modified samples. This work validates that surface energy engineering via chemical modification can effectively enhance fine particle filtration performance without compromising substrate structure.

The sixth article is also written by Weiter et al. (contribution 4), who aimed to design two mechanical modification strategies for glass fiber filter media by introducing electrospun PA66 nanofibers, and break the inherent trade-off among filtration efficiency, pressure drop, and dirt holding capacity. In Method A, nanofibers were directly blended into the glass fiber matrix at 2, 3, and 5 wt%. In Method B, nanofibers were deposited as a thin layer on the downstream side of the substrate at the same ratios. Physical characterization showed that matrix blending slightly reduced mean flow pore size and air permeability with rising nanofiber content, while surface coating dramatically decreased air permeability and minimum pore size. Filtration tests demonstrated that matrix blending significantly improved separation efficiency for fine particles (>4 μm) with negligible increase in ΔP and even enhanced DHC by up to 19%. In contrast, surface coating caused an excessive rise in ΔP and a drastic loss of DHC, making only the 2% sample testable. The optimal performance was achieved by 3% nanofiber blending, which simultaneously boosted efficiency and capacity without raising pressure drop. This study proves that internal nanofiber incorporation is a superior mechanical modification approach for high-performance depth filter media.

The seventh paper published in this Special Issue is a review by Ge et al. [30] on nature-inspired superhydrophobic textiles, summarizing their biomimetic design, fabrication strategies and practical applications. The review begins with typical superhydrophobic organisms in nature, including lotus leaves, water striders and cicada wings, and analyzes the relationship between their micro/nano hierarchical structures, low-surface-energy components and excellent water repellency. The fundamental wetting models—Young’s, Wenzel and Cassie–Baxter models—are systematically elaborated to reveal the theoretical mechanism of superhydrophobicity. The raw materials for constructing superhydrophobic textiles are classified into inorganic particles, cellulose-based and lignin-based materials, with emphasis on their biocompatibility and mechanical stability. The preparation technologies are divided into one-step methods (sol–gel, electrospinning, etching) and multi-step processes (roughness construction followed by low-surface-energy modification). Applications in self-cleaning fabrics, biomedical antifouling textiles, waterproof breathable functional materials and oil–water separation are highlighted. Finally, the challenges of complex processes, high-cost fluorine-containing reagents and poor mechanical stability are discussed, and future developments toward green, durable and scalable fabrication is prospected.

Wu et al. [31] developed a green biobased composite photocatalyst CS-LS/AgNPs by in situ reduction of silver nanoparticles on chitosan–sodium lignosulfonate carriers. The study aimed to efficiently degrade dyes in wastewater. Sodium lignosulfonate (LS) acts as both a reducing agent and a stabilizer to synthesize AgNPs without toxic chemical reductants. The structure and morphology of composites are characterized by SEM, EDS, FTIR, XRD and XPS, confirming the uniform distribution of AgNPs on CS-LS substrates. Under optimal conditions (20 mg/L RhB, 0.02 g catalyst, 60 min, 250 W UV light), the photocatalytic removal rate of RhB reaches 55%, while the degradation rates of Telon Red A2R and Direct Dark Brown ME are as high as 95% and 97%, respectively. Kinetic analysis shows that the degradation process follows the pseudo-first-order kinetic model. Radical capture experiments verify that ·O_2_^−^, ·OH and h^+^ are the main active species. The composite activity remains at 27% degradation after five cycles, showing favorable reusability. This work provides a low-cost, eco-friendly photocatalyst utilizing renewable chitosan and lignin to achieve the high-value utilization of biomass resources in dye wastewater treatment.

Wang and Liu’s study (contribution 5) focuses on the design and fabrication of high-performance hydrophobic PI/PSA/PEG nanofibrous membranes via electrospinning combined with water etching, for waterproof and moisture-permeable applications. PI and PSA are blended as the backbone matrix, and water-soluble PEG is introduced as a pore-forming agent. The effects of PEG content (mass ratio to PI/PSA from 1/1 to 1/5) on fiber morphology, mechanical properties and hydrophobicity are investigated. Under optimized conditions (15 wt% PI/PSA concentration, 10 kV voltage, PEG/PI/PSA = 1/3), the membrane shows uniform fibers with an average diameter of 0.73 μm. Water etching dissolves partial PEG, forming micro/nano rough wrinkled structures on the fiber surface. After etching, the mechanical properties are improved (breaking stress from 0.51 MPa to 1.09 MPa), and the initial water contact angle increases from 130.4° to 137.9°. The etched membrane exhibits excellent waterproofness, air permeability (46.16 mm/s) and water vapor transmission rate (4.45 kg/(m^2^·day)), as well as outstanding anti-fouling and self-cleaning functions. This green and facile strategy provides a scalable approach for constructing hydrophobic nanofibrous membranes with balanced mechanical and functional properties, showing great potential in protective textiles and waterproof breathable materials.

Tan et al.’s work [32] present a comprehensive review focusing on regenerated silk fibers, with the core target of achieving a performance comparable to natural silk fibers. This work systematically outlines the superior mechanical properties of natural spider silk and silkworm silk, along with the bottlenecks of low yield and difficulty in large-scale breeding. The primary, secondary, and spatial structures of silk fibroin (SF) are elaborated, including the heavy chain/light chain/P25 subunits, as well as the α-helix, β-sheet, β-turn, and random coil conformations. Characterization methods such as FTIR, Raman, XRD, and NMR for analyzing SF secondary structures are summarized. The natural eco-spinning process of silkworms is highlighted, emphasizing the liquid crystal mechanism and the conformational transition from random/α-helix to β-sheet under shear and metal ion effects. Various bionic spinning strategies are reviewed, covering solvent systems (neutral salts, ionic liquids, acids), spinning methods (wet spinning, dry spinning, dry-jet wet spinning), and the exploration of liquid crystal spinning. Current challenges, including structural damage during dissolution, insufficient mechanical properties of regenerated fibers, and process limitations, are discussed. This review provides theoretical guidance for developing high-performance regenerated protein fibers that match natural silk.

## 3. Conclusions

In summary, the research explored in this Special Issue indicates that advanced textile materials have entered a new era of intelligence, multifunctionality, green production, and scalable manufacturing. These studies cover biomimetic design, flexible electronics, zero-energy thermal management, and multi-scenario protection, as well as advancements from laboratory to industrial processing, reflecting the forefront direction and core competitiveness of this field. Future research will prioritize four key directions: ultra-fast response and highly stable adaptive intelligent textiles; green, low-cost, large-scale manufacturing; standardized evaluation of washability, durability, and biocompatibility; and its wide applications in biomedical, aerospace, building energy efficiency, and seawater desalination fields. This Special Issue comprehensively updates the most advanced textiles, aiming to stimulate innovation, bridge interdisciplinary cooperation, accelerate the transformation from laboratory to market, and ultimately promote industrial upgrading towards a healthier, safer, low-carbon, and smarter future.
